# Exploring the uncommon: Unusual instance of retained fractured needles in a patient of intravenous drugs abuse

**DOI:** 10.1016/j.radcr.2024.01.016

**Published:** 2024-02-05

**Authors:** Tejas Phaterpekar, Muhammad Israr Ahmad, Hugue Ouellete, Peter Munk, Paul Mallinson, Savvas Nicolaou, Adnan Sheikh

**Affiliations:** a Faculty of Medicine, The University of British Columbia, Vancouver Campus |Musqueam, Squamish & Tsleil-Waututh Traditional Territory], Vancouver, BC, Canada; b Radiology Department, University of British Columbia, Vancouver, British Columbia, Canada

**Keywords:** IV Drug abuse complications, Fracture needles, Foreign bodies, Recurrent infections, Sepsis risk

## Abstract

Retained needle fragments commonly serve as sources of recurrent infections with a potential to embolize to the heart and lungs and can lead to life-threatening consequences. Here, we report a case of a 46-year-old male with a history of intravenous drug user and chronic forearm wounds, presenting with sepsis. Several retained needles are identified on CT scan, several days postadmission. This case highlights the importance of timely assessment of infectious sources in patients with history of intravenous drug abuse.

## Introduction

Retained needles are a common occurrence in intravenous drug user (IVDU) patients, yet they are an understudied and underassessed aspect of patient care. One study found that up to 20% of IVDU patients had experienced breakage of their needles at least once during their usage history [1]. Of these breakage events, a majority involved reusing needles in popular sites along the arms and legs. While broken needles can be recovered through surgical intervention, or by direct action of the individual, needles can be retained for lengthy periods of time and can serve as a nidus for infection, abscess formation, and embolize to other locations in the body [2]. Despite infectious complications accounting for 60%-80% of IVDU patient hospital admissions per year [3], history-taking, and assessment around retained needles remains limited.

We present a case of a 46-year-old male, with a history of IVDU, presenting with several retained needles identified several days after admission for bacteremia and infective endocarditis.

## Case description

A 46-year-old male was admitted to our hospital with a 3-day history of fever, nausea, vomiting, lethargy, confusion, and increased work of breathing. For the past month, he reported experiencing bilateral arm pain, erythema, and swelling, however he had not accessed care for antibiotics. His past medical history included opioid use disorder, microcytic anemia, anasarca, and chronic bilateral forearm wounds due to repeated and ongoing IV drug abuse.

On presentation, the patient had decreased air entry bilaterally, subtle crackles, and a harsh pansystolic murmur at the upper sternal border. A Focused Assessment with Sonography for Trauma (FAST Scan) did not detect any free fluid or pericardial effusion. Large ulcerations measuring 10 × 5 cm were noted on his forearms with purulent drainage. Significant pitting edema was also noted in all 4 extremities, consistent with anasarca.

Work up in the Emergency Department revealed leukocytosis (28.0 × 10^9^/L) with neutrophilia (90%), thrombophilia (521 × 10^9^ /L), and severe microcytic anemia (Hb 50 g/L, MCV 63 fL). Blood cultures showed initial methicillin susceptible staphylococcus aureus bacteremia, and wound cultures of the left arm grew group B streptococcus. Despite elevated troponin (2419 ng/L) and B-Type natriuretic peptide (9769 ng/L), an electrocardiogram was unremarkable for type I cardiac ischemia. A CT Pulmonary Angiogram was also ordered which demonstrated a small right pleural effusion but no evidence of pulmonary embolism.

**Fig. 1:**
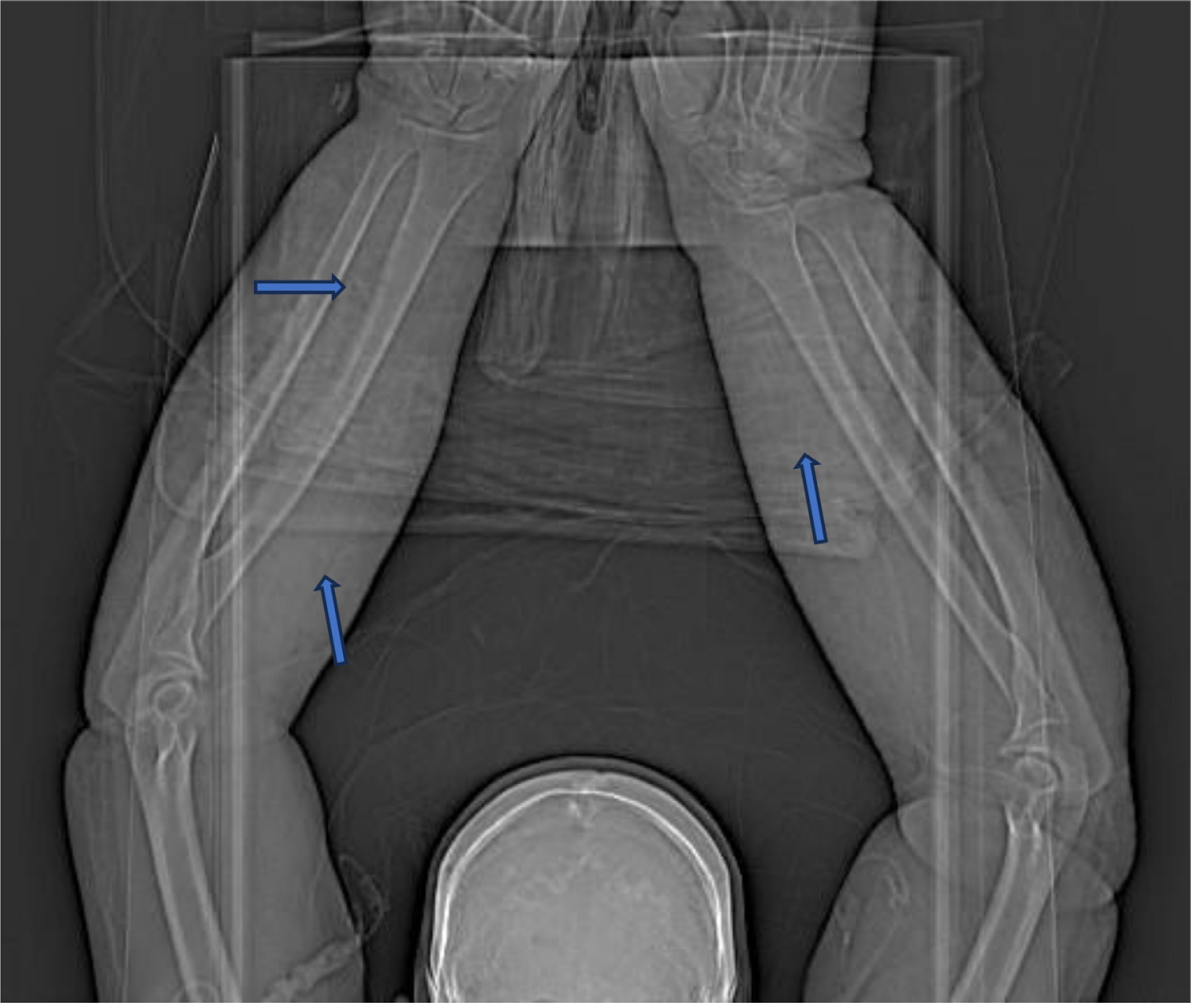
CT Scanogram demonstrating several linear high densities (blue arrows).

**Fig. 2:**
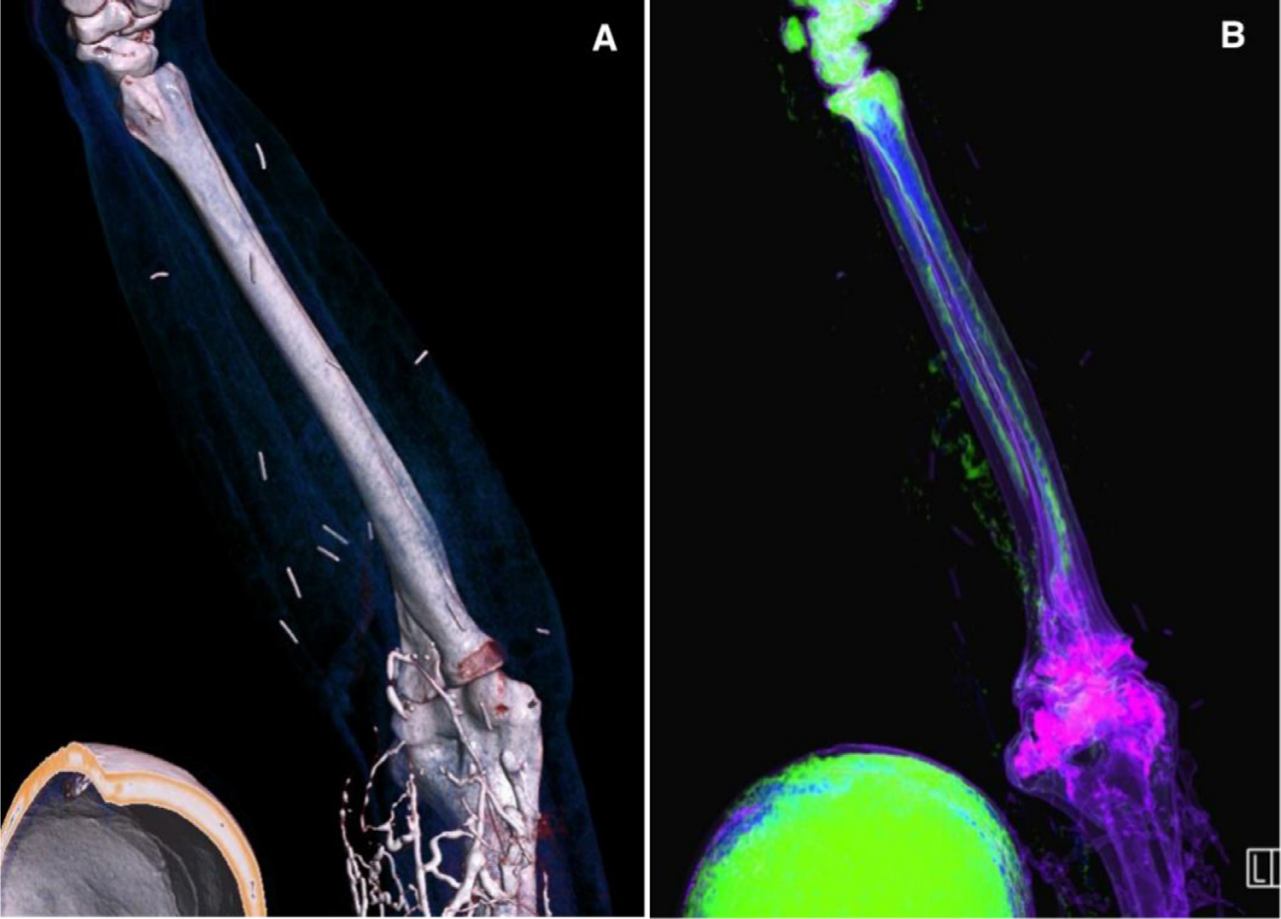
3D reformates demonstrating multiple linear objects in the forearms bilaterally (A and B). These linear objects had a uniform length and were different in density as compared to mild retrograde contrast reflux in the veins closer to the site of contrast administration.

**Fig. 3:**
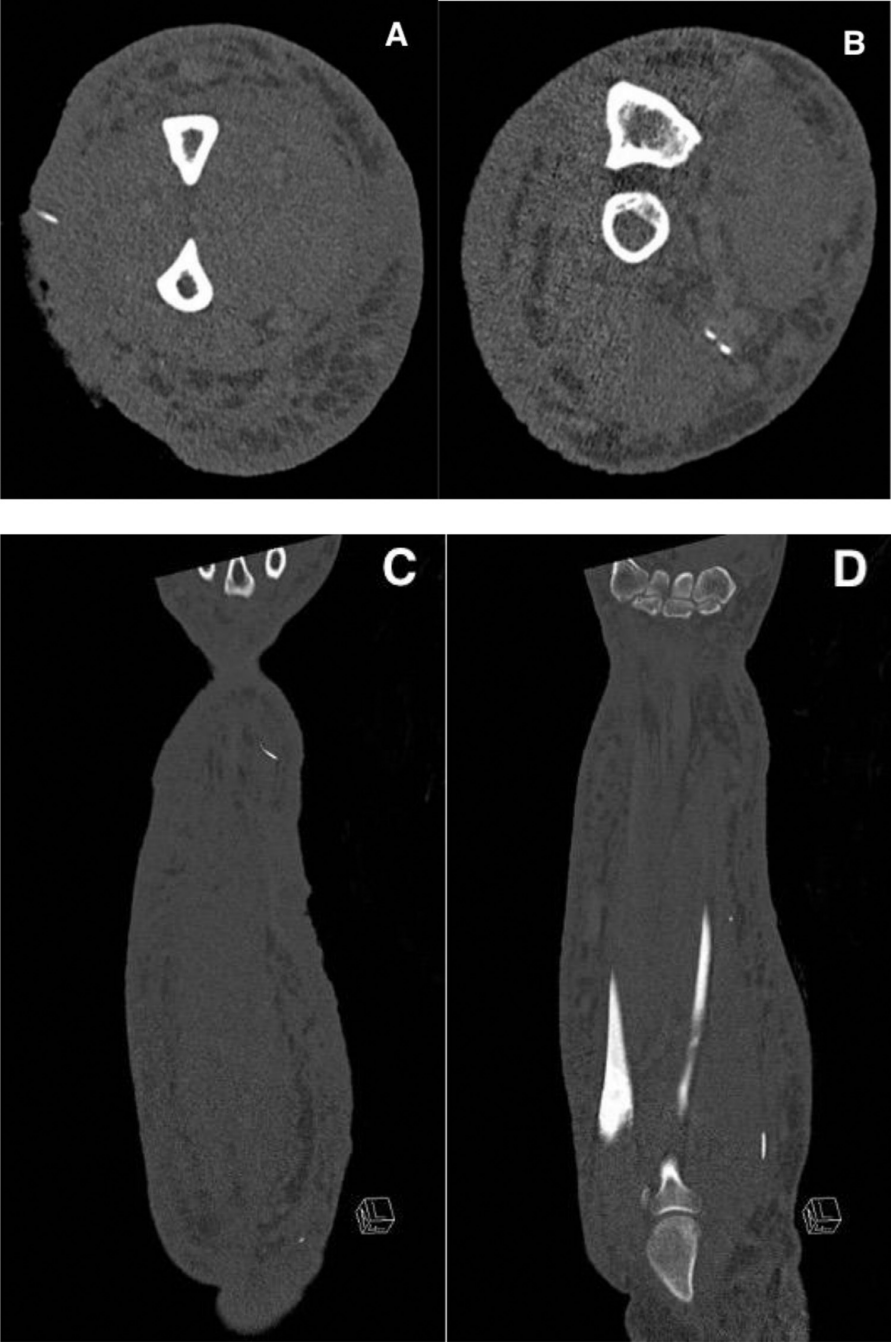
Contrast enhanced CT – Axial (A and B) and coronal (C and D) reformats demonstrating superficial and deep-seated uniform sized linear high-density objects, in keep with retained foreign bodies / needles related to IVDU along with area of skin ulceration. No abcess or osseous destruction was seen. Diffuse anasarca noted.

While an initial transthoracic echocardiogram identified no vegetation, a coronary CT (Computed Tomography) angiogram was performed 5 days postadmission, showing a 10 mm hypodense lesion arising from the aortic valve leaflets. This was later confirmed to be a vegetation with a subsequent transesophageal echocardiogram. On the same day, a bilateral forearm CT with contrast was done to assess for any drainable collections (Fig. 1, Fig. 2-3), none were identified, however multiple high-density linear foci were seen within the subcutaneous and deep soft tissues bilaterally, consistent with retained needles. Fortunately, there was no evidence of acute osseus lesions or joint effusions.

After initial evaluation, the patient was admitted and followed by departments of Infectious Diseases, Internal Medicine, and Cardiology. He responded well to broad spectrum antibiotics (Piperacillin-Tazobactam and Vancomycin), 2 units of packed red blood cells, and several days of IV furosemide. Cardiac surgery was also consulted regarding the suspected aortic endocarditis and opted towards medical management in the absence of aortic regurgitation. While in hospital, Plastic surgery team assessed the necessity of removing the foreign bodies. Due to the lack of pain, sensory and functional defects operative management was not recommended. His repeat blood cultures cleared at 4 days postadmission, and he remained clinically stable throughout his total stay of 13 days.

He was switched to IV Cefazolin during his hospital stay, and continued treatment for 6 weeks as an outpatient with follow up by infectious diseases. His forearm wounds had essentially healed by the time he was assessed by Plastic surgery as an outpatient 1 month later.

## Discussion

In this case, a 46-year-old male, with a history of IVDU and chronic forearm wounds, presents with bacteremia and infective endocarditis. Acute resuscitation was carried out successfully, but identification of retained needles did not take place until several days after his initial presentation, despite knowledge of his intravenous drug use.

Beyond infection, retained needles have the potential to embolize at any time, depending on several factors including needle shape, size, as well as blood flow [2]. Dangerous embolization sites like the heart and lungs put patients at risk for severe consequences like pericarditis, endocarditis, and pulmonary abscess. In 1 case, a 40-year-old male IVDU patient experienced needle breakage in his right groin, and it was later located in his RV, ultimately resulting in the need for open heart surgery [4]. In another case, a 22-year-old female presented with a fully embedded needle fragment in her right ventricle, that was nonoperable and predisposing of future endocarditis [5]. Finally, a 36-year-old IVDU patient presenting with a resultant intrapulmonic abscess after experiencing breakage of needles in his neck [6]. Fortunately, the retained needles in our case did not involve vessels like seen in other cases [7]. Nonetheless, these cases demonstrate some of the rare, but dangerous, downstream consequences of retained needles, necessitating earlier assessment, and prevention of these events.

Retained needles can be challenging to identify due to their small dimensions, in the context of a clinical history that can be unreliable [8]. For superficial soft tissues, ultrasonography is well established in identifying radiolucent foreign bodies, and has comparable efficacy to CT in identifying radiopaque foreign bodies, while being timelier and more cost-effective. One recent case demonstrated the clinical use of Point-Of-Care Ultrasound in identifying a retained needle that penetrated through a patient's left superficial and deep femoral arteries [9]. The patient was taken directly from the emergency department to the operating room and the needle was removed with no complications.

In our case, initial assessment was cardiac-focused, given his elevated cardiac markers, anasarca, and initial chest X-ray findings. However, a brief point-of-care ultrasound assessment of his injection sites after initial stabilization seems reasonable, especially given his recent drug use, chronic forearm wounds, and septic clinical picture. Early imaging would have provided information on potential sources of infection and allowed for earlier intervention if any of the needles were dangerously located and warranted operative management.

Unfortunately, many barriers in accessing care exist for the IVDU patients. If the patient had received healthcare earlier for his chronic bilateral forearm wounds, it is entirely possible the hospital admission could have been avoided. Furthermore, there remains an increased need of bringing awareness of retained needles to the attention of healthcare professionals and patients. Many individuals in these cases report felt the needle breaking during use. History-taking that explicitly focuses on needle breakage could result in earlier suspicion for needle retention.

## Conclusion

Needle retention in IVDU patients can lead to life-threatening consequences if not recognized in a timely manner. Here we suggest the benefit of imaging of the injection sites for IVDU patients presenting with sepsis, to identify hidden sources of infection and guide operative removal of dangerously located retained needles. By increasing awareness of cases like these, we hope to bring attention to the serious complications that can occur if retained needles are not investigated.

## Patient consent

A written informed consent was obtained from the patient for the purpose of this case report.

## References

[1] Norfolk GA, Gray SF (2003). Intravenous drug users and broken needles–a hidden risk?. Addiction.

[2] Kulaylat MN, Barakat N, Stephan RN, Gutierrez I (1993). Embolization of illicit needle fragments. J Emerg Med.

[3] Theodorou SJ, Theodorou DJ, Resnick D (2008). Imaging findings of complications affecting the upper extremity in intravenous drug users: featured cases. Emerg Radiol.

[4] Fu X, Chen K, Liao X, Shen K (2017). Case report: surgical removal of a migrated needle in right ventricle of an intravenous drug user. Subst Abuse Treat Prev Policy.

[5] Ngaage D, Cowen M (2001). Right ventricular needle embolus in an injecting drug user: the need for early removal. Emerg Med J.

[6] Angelos MG, Sheets CA, Zych PR (1986). Needle emboli to lung following intravenous drug abuse. J Emerg Med.

[7] Gladman J (2019). Pins and needles in the groin: an incidental finding of retained needle fragments in an intravenous drug user. BMJ Case Rep.

[8] Contegiacomo A, Conti M, Trombatore P, Dezio M, Muciaccia M, Lozupone E (2020). Radiological features and management of retained needles. Br J Radiol.

[9] Primi B, Thiessen MEW (2016). Point-of-care ultrasound to locate retained intravenous drug needle in the femoral artery. West J Emerg Med.

