# Corrigendum to “Exploring the uncommon: Unusual instance of retained fractured needles in a patient of intravenous drugs abuse” [Radiology Case Reports 19 (2024) 1619-1623]

**DOI:** 10.1016/j.radcr.2024.02.096

**Published:** 2024-03-21

**Authors:** Tejas Phaterpekar, Muhammad Israr Ahmad, Hugue Ouellette, Peter Munk, Paul Mallinson, Savvas Nicolaou, Adnan Sheikh

**Affiliations:** a Faculty of Medicine, The University of British Columbia, Vancouver Campus |Musqueam, Squamish & Tsleil-Waututh Traditional Territory], Vancouver, BC, Canada; b Radiology Department, University of British Columbia, Vancouver, British Columbia, Canada

The authors would like to inform you that the name of the third author, Hugue Ouellette, was misspelt in their published article. The correct spelling is provided here.

